# A Randomized Controlled Trial of Botulinum Toxin Combined with Non-Cross-Linked Bovine Collagen for Improving Periorbital Wrinkles

**DOI:** 10.1007/s00266-025-04906-9

**Published:** 2025-08-13

**Authors:** Yao Liu, Lin Wang

**Affiliations:** Central Hospital of Dalian University of Technology (Dalian Municipal Central Hospital), Dalian, 116038 Liaoning China

**Keywords:** Botulinum toxin type A, Collagen filler, Periorbital wrinkles, Facial cosmetology

## Abstract

**Background:**

This study examined the clinical efficacy of botulinum toxin type A (BTX-A) combined with non-cross-linked bovine collagen in improving periorbital wrinkles.

**Methods:**

Forty participants with periorbital wrinkles were recruited from the Plastic Surgery and Aesthetic Department of the Central Hospital Affiliated to Dalian University of Technology in China between November 2021 and August 2022. Participants were randomly assigned to either an observation group or a control group, with each group comprising 20 participants. The control group received BTX-A injection alone, whereas the observation group received combined injections of BTX-A and collagen filler.

**Results:**

Standardized photographic images were taken at baseline and at 1-week, 1-month, 3-month, and 6-month follow-ups after the injection. The rejuvenation effect was evaluated using the 5-grade Wrinkle Severity Rating Scale, whereas patient satisfaction was assessed using the Global Aesthetic Improvement Scale. Both groups achieved a total effective rate of 100%. However, the markedly effective rate was significantly higher in the observation group (90%) than in the control group (40%; *p *< 0.01). Moreover, 95% patients in the observation group reported great satisfaction, and the percentage was significantly higher than that in the control group (20%; *p *< 0.01). No adverse events were reported during the study.

**Conclusion:**

BTX-A injections combined with non-cross-linked bovine collagen filler provide a highly effective and safe therapy for improving periorbital wrinkles.

**Level of Evidence II:**

This journal requires that authors assign a level of evidence to each article. For a full description of these Evidence-Based Medicine ratings, please refer to the Table of Contents or the online Instructions to Authors  www.springer.com/00266.

## Introduction

As individuals age, various factors contribute to the gradual aging of the skin. Periorbital wrinkles are among the earliest and most noticeable facial wrinkles, including features such as blepharoptosis, lower eyelid sagging, crow’s feet, glabellar frown lines, forehead wrinkles, and bunny lines. In severe cases, these changes may be accompanied by the loss of tissue volume in the upper and lower eyelids, depression, pouch formation, and tear trough deformities [1]. To address periorbital aging, various interventions are available, including surgical procedures, skin resurfacing, chemical peels, laser ablation, radiofrequency treatments, dermal filler injections, and neuromodulation using botulinum toxin [1]. The primary contributors to periorbital wrinkles are muscular hyperactivity and volume loss, which together exacerbate the signs of aging. Nonsurgical approaches, particularly minimally invasive injection therapies, have gained popularity as the preferred treatment options [2, 3]. The combined application of botulinum toxin type A (BTX-A) and collagen offers a dual mechanism to correct wrinkles by restoring skin volume and relaxing muscle activity simultaneously. This study employed a variety of professional instruments to assess changes in periorbital wrinkles and skin characteristics before and after treatment, aiming to provide an objective evaluation of the clinical efficacy of BTX-A injections combined with collagen fillers.

## Materials and Methods

### Design and Participants

This prospective clinical study recruited 40 participants with periorbital wrinkles from the Central Hospital Affiliated to Dalian University of Technology. The sample comprised 39 female participants and 1 male participant, aged 32–56 years, with an average age of 40 years. The 40 participants were randomized into either a control group or an observation group by using a lot-drawing method, with 20 individuals in each group. No significant differences were observed in baseline characteristics between the two groups (*p *> 0.05). Patients meeting the following criteria were included in the study: (i) healthy male and female individuals aged 30–65 years; (ii) presence of bilaterally symmetric moderate (3) or severe (4) periorbital wrinkles, as assessed using the Wrinkle Severity Rating Scale (WSRS) [4] prior to the procedure; (iii) willingness to improve periorbital wrinkles, with Fitzpatrick skin types III and IV [5]; and (iv) stable lifestyle and work commitments over the preceding 6 months, with the ability to attend regular follow-ups. Exclusion criteria included a history of scar-prone skin, systemic diseases or tumors, severe infectious diseases, coagulation dysfunction, allergies to botulinum toxin or lidocaine, local skin damage, or other active skin conditions such as infections or herpes simplex. Additional exclusion criteria were pregnancy or plans for lactation within 6 months, high sensitivity to sunlight, inability to attend follow-up visits as scheduled, and unrealistic expectations of therapeutic outcomes. All participants provided signed informed consent before their enrollment in the study.

### Treatment Procedure

**Fig. 1:**
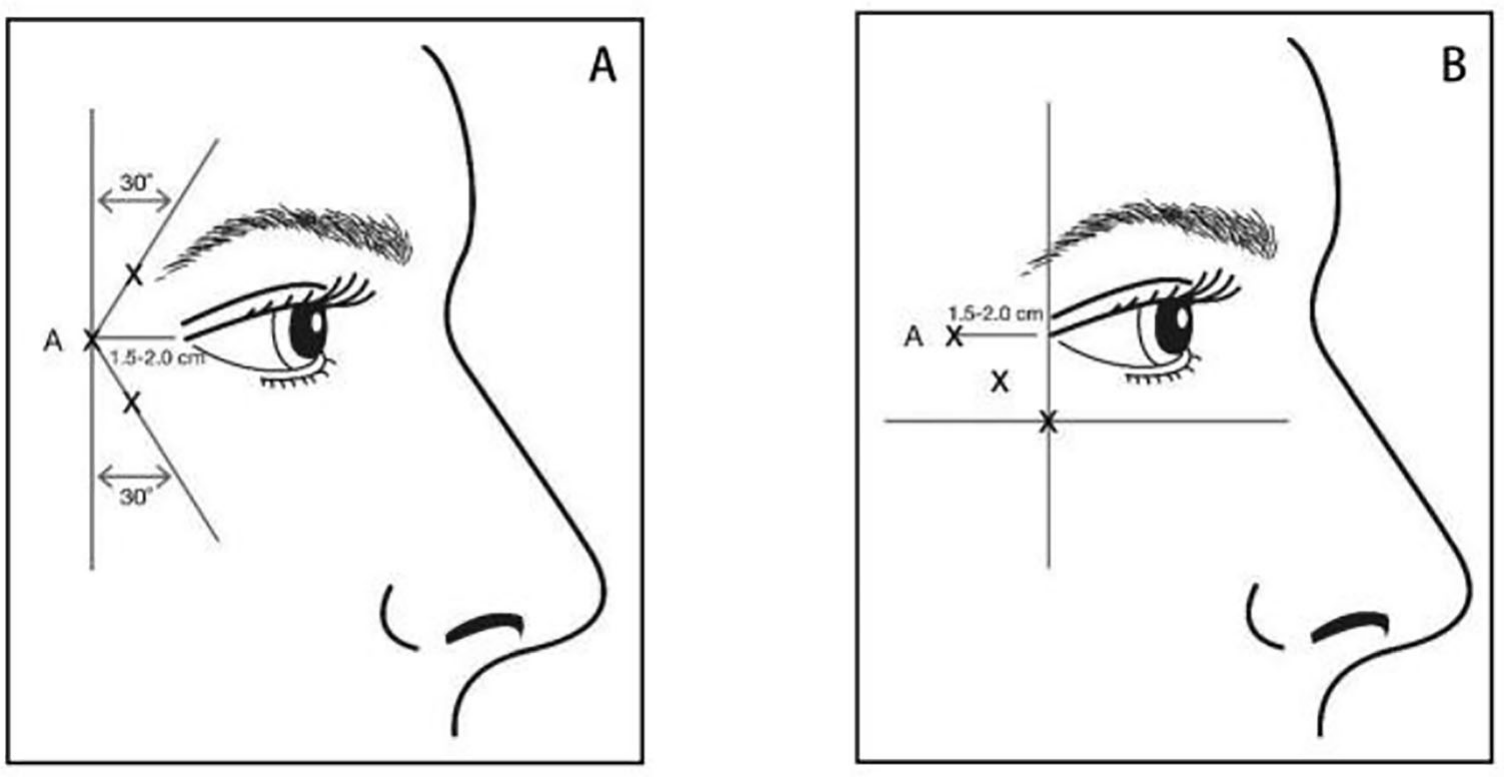
Botulinum toxin injection site. Picture quoted from the label of botulinum toxin type A for injection

Control group: In this group, periorbital wrinkles (crow’s feet) were treated by administering the injection of botulinum toxin type A (Botox, Allergan Pharmaceutical Company, USA; 100-U/vial; imported drug registration number: S20070023) alone. A 100-U vial of botulinum toxin was diluted with 2.5 mL of 9% sterile saline solution. The solution was then injected into the lateral orbicularis oculi muscle by using a 30G needle, targeting three injection sites on each side (a total of six injection sites). The total administered dose was 24 U/0.6 mL, with 12 U per side. For wrinkles primarily located above and below the lateral canthus, the first injection site was marked approximately 1.5–2.0 cm temporal to the lateral canthus and lateral to the orbital rim. Two additional sites were marked: one at 1.0–1.5 cm above the first site and the other at an approximate 30° medial angle (Fig. 1A). For crow’s feet predominantly located below the lateral canthus, the injection sites were angled from anteroinferior to superoposterior, as shown in Fig. 1B. After the injections, the local area was compressed for 1–5 min to minimize swelling and bruising.

Observation group: Static wrinkles in the periorbital region were marked prior to the treatment. After the application of surface anesthesia, collagen filler (Fillderm, Fiman (Changchun) Pharmaceutical Biotechnology Company Limited; national medical device approval: 20163131609) was used to fill the static wrinkles. Depending on the wrinkle depth, the collagen injection dose was 0.1–0.15 mL per centimeter of wrinkle length, with a total volume of 0.5–0.8 mL for both sides. Collagen was injected into the middle and deep layers of the dermis by using a 30G sharp needle, starting at the outer edges of the wrinkles. The injection sequence followed a pattern from the outer to inner regions and from the deeper to more superficial layers. The principle of “deep wrinkles are filled superficially, and mild wrinkles are filled deeply” was applied to achieve immediate filling of fine lines and depressions. Following collagen injection, BTX-A injection was administered using the same method and in the same dosage as described for the control group. Seven days after the initial treatment, the administering physician evaluated the treated areas. Regions where the best filling effect had not been achieved and static wrinkles persisted were marked and administered a second supplementary collagen injection. All injections involved in this study were performed by the same designated chief physician of the department.

### Measurements

#### Digital Photography

Standardized frontal, 45°, and lateral view photographs were taken at baseline (before injections) and during follow-up visits by using a Canon G9X digital camera (Canon, Tokyo, Japan). These photographs were used to assess wrinkle severity by using the 5-grade Wrinkle Severity Rating Scale (WSRS; Table 1) at baseline and at 1-week, 1-month, 3-month, and 6-month follow-ups after injection. Assessments were conducted by more than two plastic surgeons qualified as associate chief physicians or above. The evaluation criteria were defined as follows: treatment was considered markedly effective if the wrinkle severity after injection showed a reduction of ≥ 2 grades compared with the baseline, effective if there was a reduction of ≥ 1 grade, and ineffective if there was no change in wrinkle severity (a difference of 0 grades).

**Table 1:** Wrinkle severity rating scale [4]

Grade	Definitions
I	Absent: no visible wrinkle; continuous skin line
II	Mild: shallow but visible wrinkle with a slight indentation; minor facial feature
III	Moderate: moderately deep wrinkle, clear facial feature visible at normal appearance but not when stretched
IV	Severe: very long and deep wrinkle, prominent facial feature; < 2 mm visible fold when stretched
V	Extreme: extremely deep and long wrinkle, detrimental to facial appearance; 2–4 mm visible V-shaped fold when stretched

$$\text{Effective rate}=\frac{\text{Markedly effective}+\mathrm{Effective}}{\text{Totally cases}}\times 100\mathrm{\%}$$

#### Participant Satisfaction

Using a satisfaction rating scale, evaluations were conducted at 1 week, 1 month, 3 months, and 6 months after the treatment. Participants’ satisfaction was assessed using the Global Aesthetic Improvement (GAIS) score, where Level 1 indicates “very satisfied,” Levels 2 and 3 indicate “satisfied,” and Levels 4 and 5 indicate “dissatisfied” (Table 2).

**Table 2:** Global beauty improvement (GAIS) score

Grade	Graded global beauty effect
Level I	Improvement is very significant
level II	Significant improvement
Level III	A certain degree of improvement
Level IV	Remains unchanged
Level V	Worse than before

#### CK Analysis

The values of skin moisture content and elasticity were measured at baseline and at 1-week, 1-month, 3-month, and 6-month intervals using the Cutometer dual MPA580 (Courage & Khazaka Electronic GmbH, Cologne, Germany) [6], a noninvasive instrument designed for assessing skin physiological properties. Data collected before and after the treatment were compared and analyzed.

#### ThinkView Detection

Wrinkle length was measured using the ThinkView facial imaging analyzer (Taiwan Boshi Electronics Co., Ltd) at baseline and at 1-week, 1-month, 3-month, and 6-month intervals.

#### Assessment of Safety

Adverse reactions were monitored and documented, with a specific evaluation conducted based on their severity.

#### Statistical Analysis

Data analysis was performed using SPSS (version 23.0; SPSS Inc., Chicago, IL, USA). Statistical significance was determined using a Chi-square test, with a *p* value of < 0.05 considered statistically significant.

## Results

### Baseline Characteristics

Forty adult participants were recruited in this study. 39 enrolled participants (1 men and 38 women, aged 40 ± 3) completed the study and participated in the 6-month follow-up. The predominant skin types of them were Fitzpatrick skin type III (56.4%), and the majority had wrinkle severity classified as grade III (56%). The demographic characteristics are presented in Table 3.

**Table 3:** Baseline characteristics of participants

Parameters	Total (n = 39)
Age (years), mean±SD	40 ± 3
Age range (years)	34–45
Gender, n (%)	
Female	38 (97)
Male	1 (3)
Wrinkle severity, n (%)	
III	22 (56)
IV	17(44)
Fitzpatrick skin type, n (%)	
III	22 (56)
IV	17 (44)
Pain score, mean±SD	2.0 ± 1.6
The range of pain scores	(0–6)

### Patient Self-Assessment

The total patient satisfaction rate in both groups was 100%. However, the rate of great satisfaction in the observation group was 95%, significantly higher than that of 21% observed in the control group (Table 4). The difference in the satisfaction rate between the two groups was statistically significant (*p *< 0.01).

**Table 4:** Customer satisfaction evaluating

Group	Great satisfaction n (%)	Satisfaction n (%)	Dissatisfaction n (%)
Control (n = 19)	4 (21)	15 (79)	0 (0)
Observation (n = 20)	19 (95)	1 (5)	0 (0)

### Investigator Assessments

The total effective rate was also 100% in both groups. The rate of markedly effective outcomes in the observation group was 90%, significantly higher than the 42% reported in the control group (Table 5), with the difference between the two groups being statistically significant (*p *< 0.01).

**Table 5:** Comparison of the effective rate

Group	Markedly effective n (%)	Effective n (%)	Ineffective n (%)	Total effective rate %
Control(n = 19)	8 (42)	11 (58)	0 (0)	100
Observation (n = 20)	18 (90)	2 (10)	0 (0)	100

**Fig. 2:**
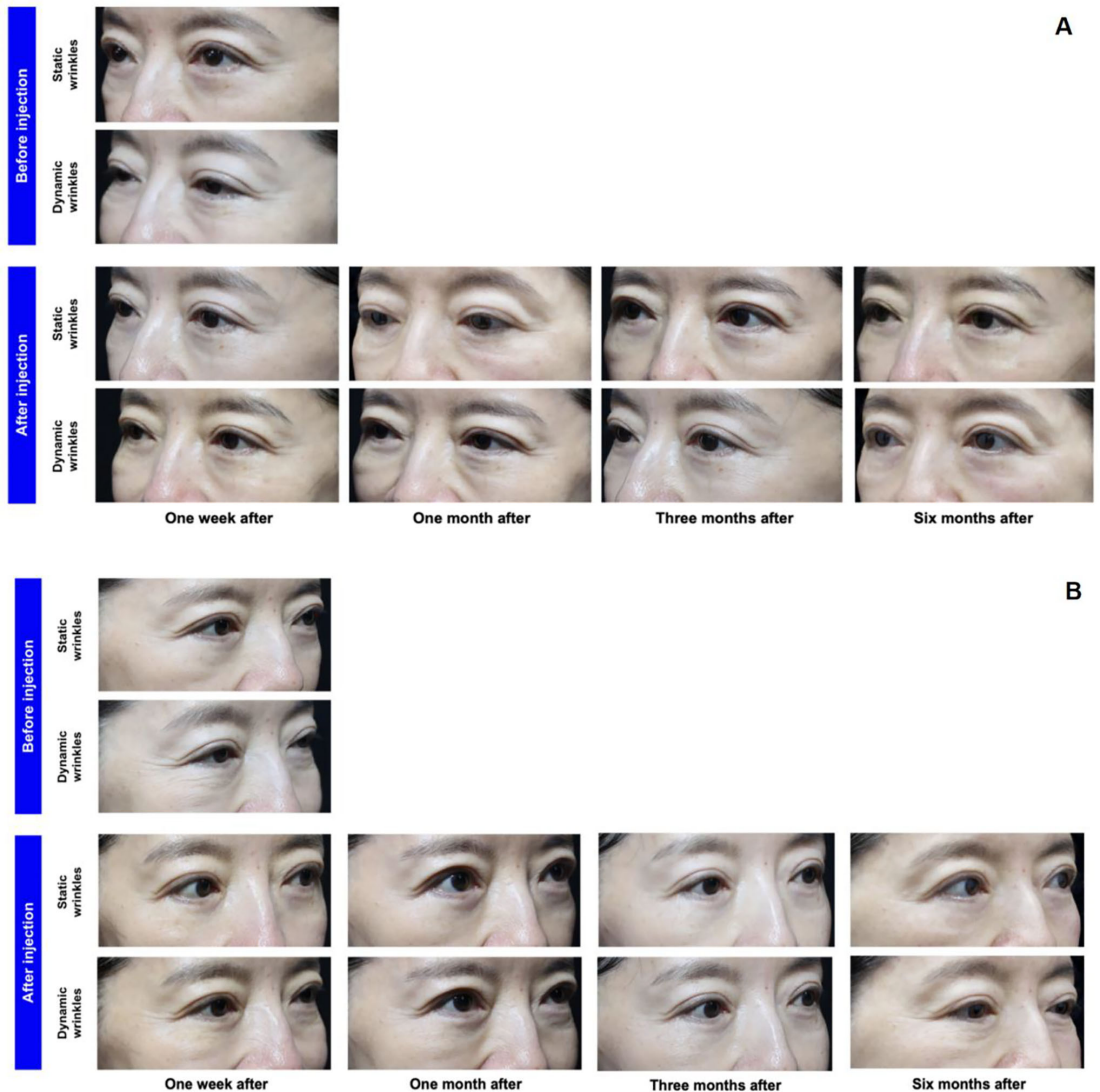
Clinical photographs of a 42-year-old woman with grade IV severity assessed using the Wrinkle severity rating scale. Changes in periorbital wrinkles before and after BTX-A combined with collagen treatment on her left side (**A**) and right side (**B**) are shown.

Clinical photographs taken before and after the treatment demonstrated that the combination of BTX-A and collagen injections resulted in noticeably shallower and shorter fine lines in the periorbital region on both sides of the participants, with the most pronounced improvement observed at the 3-month follow-up. Although the effect diminished over time, it remained a significant improvement compared to the condition at baseline (Fig. 2). Similar trends were observed in the control group, with the best effect also noted at the 3-month follow-up.

**Fig. 3:**
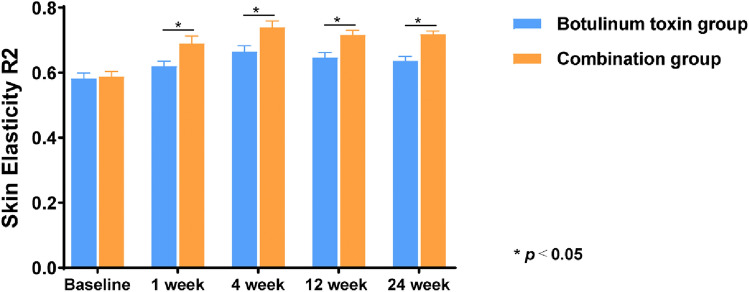
Comparison of the skin moisture content before and after injection

**Fig. 4:**
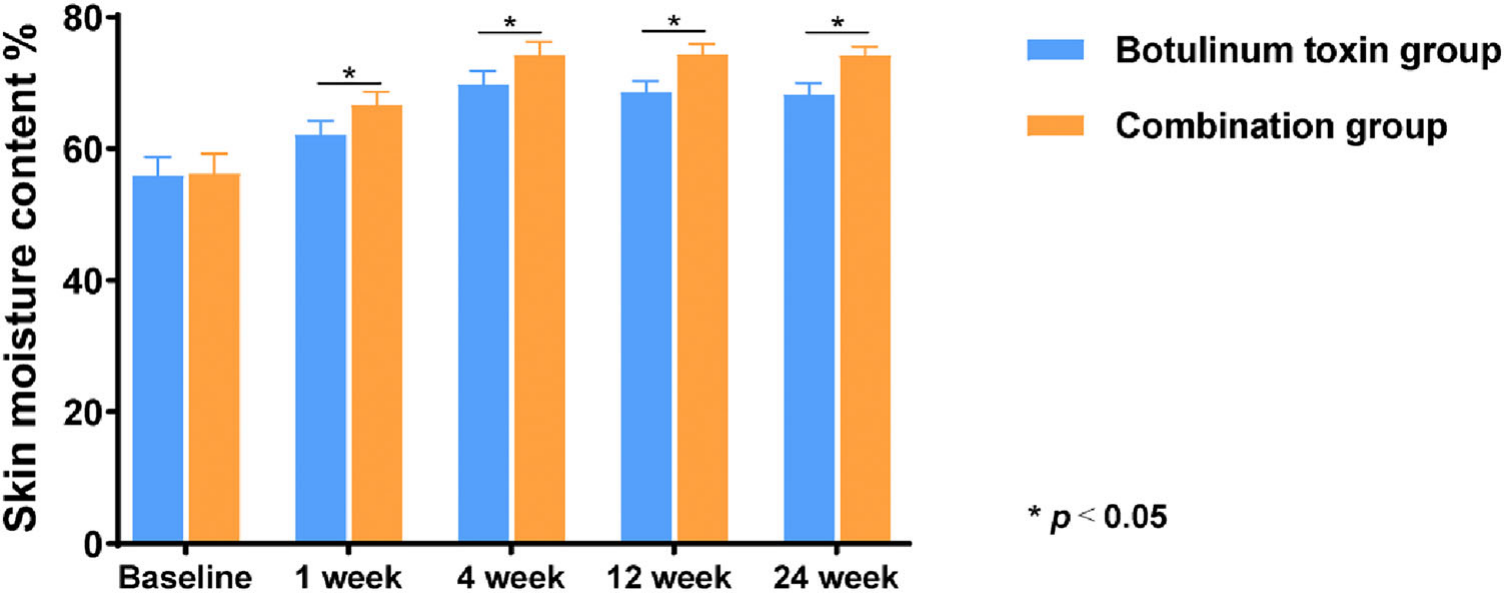
Comparison of skin elasticity before and after injection

Changes in skin elasticity and moisture content were measured using noninvasive instruments. As shown in Fig. 3, the results indicated that 1 month after injection, the improvements in skin elasticity and moisture content in the observation group were significantly greater than those in the control group (*p *< 0.05). The combined use of collagen and BTX-A injection led to a marked enhancement in the elasticity of the periorbital skin (Fig. 4).

**Fig. 5:**
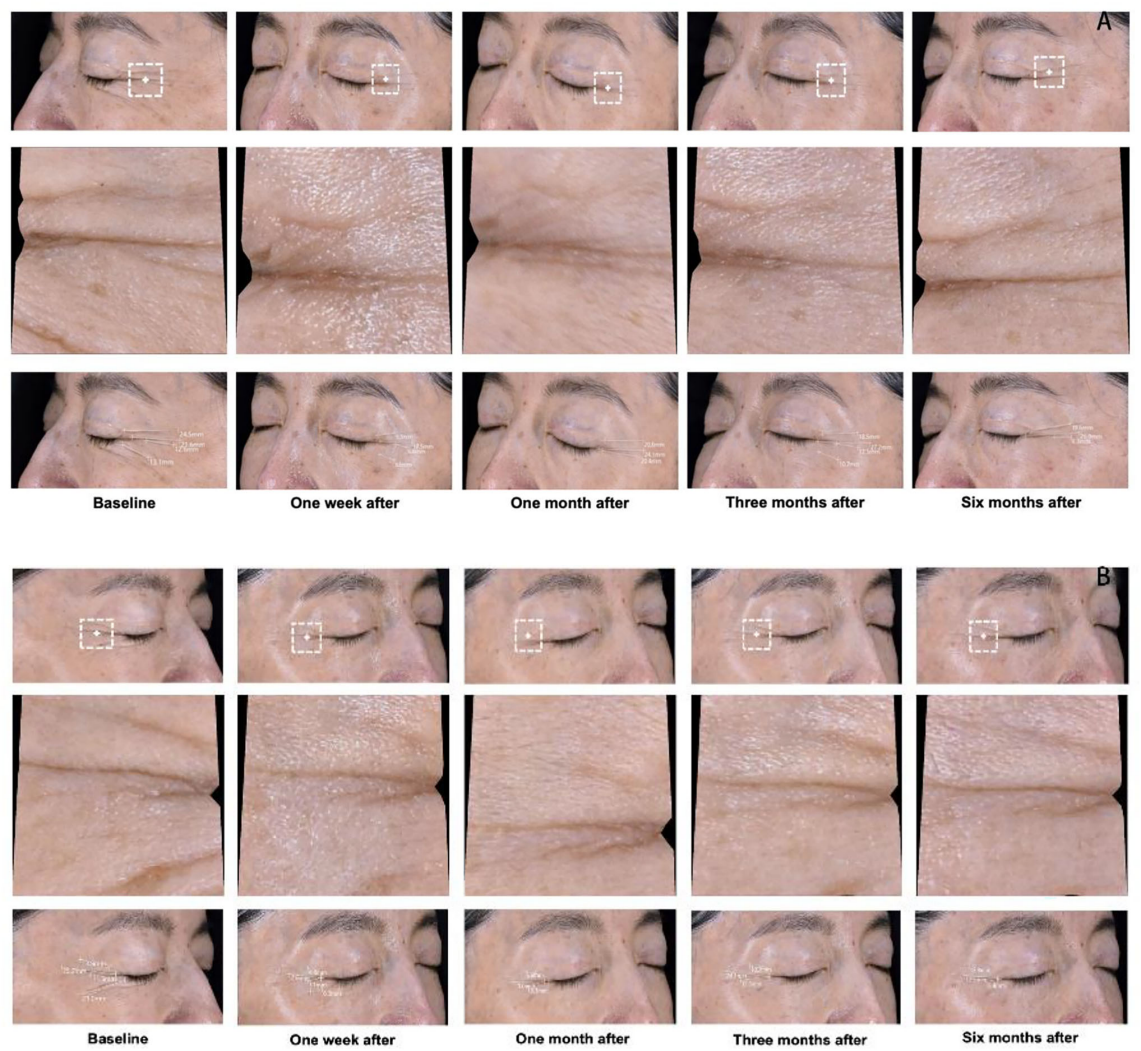
Dynamic changes in wrinkles after the injection of BTX-A combined with collagen on the left and right sides (**A** and **B**), assessed using ThinkView.

Changes in periorbital wrinkles were objectively analyzed using the ThinkView system. One week after injection, the length and depth of wrinkles showed significant reduction, with the most notable improvements observed at the 3-month follow-up (Fig. 5).

**Fig. 6:**
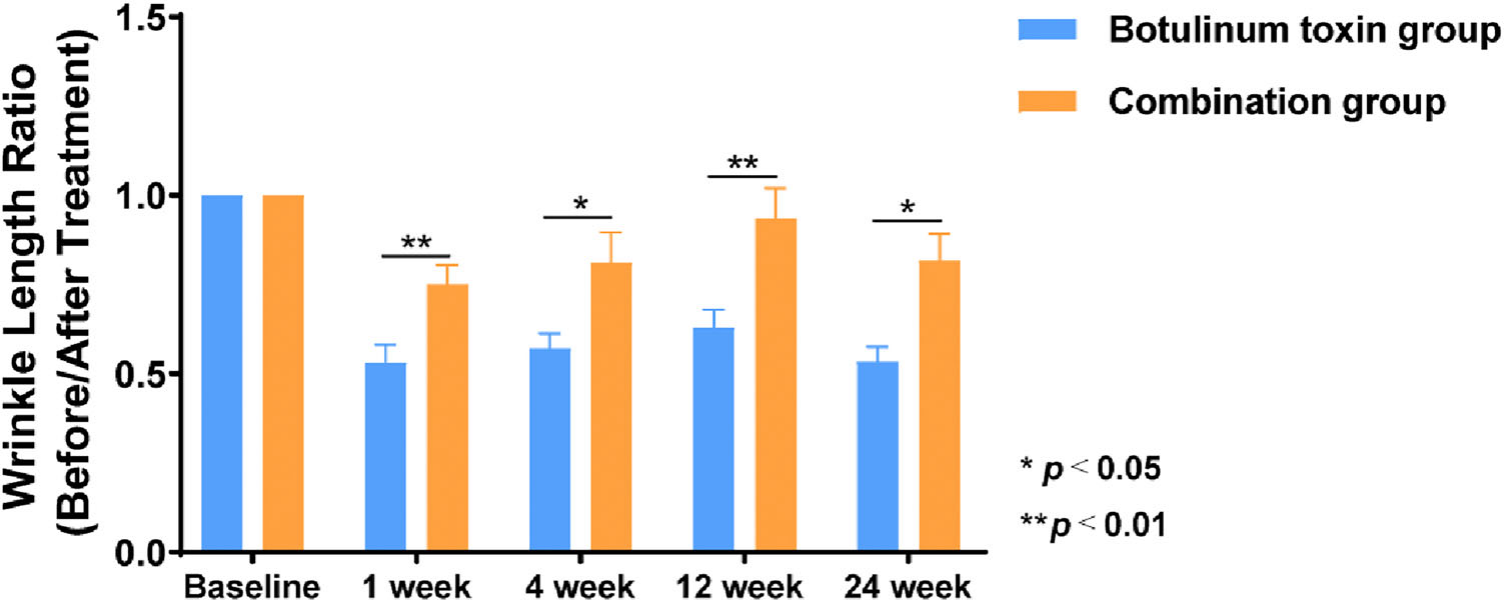
Comparison of the ratio of wrinkle length before and after injection

**Fig. 7:**
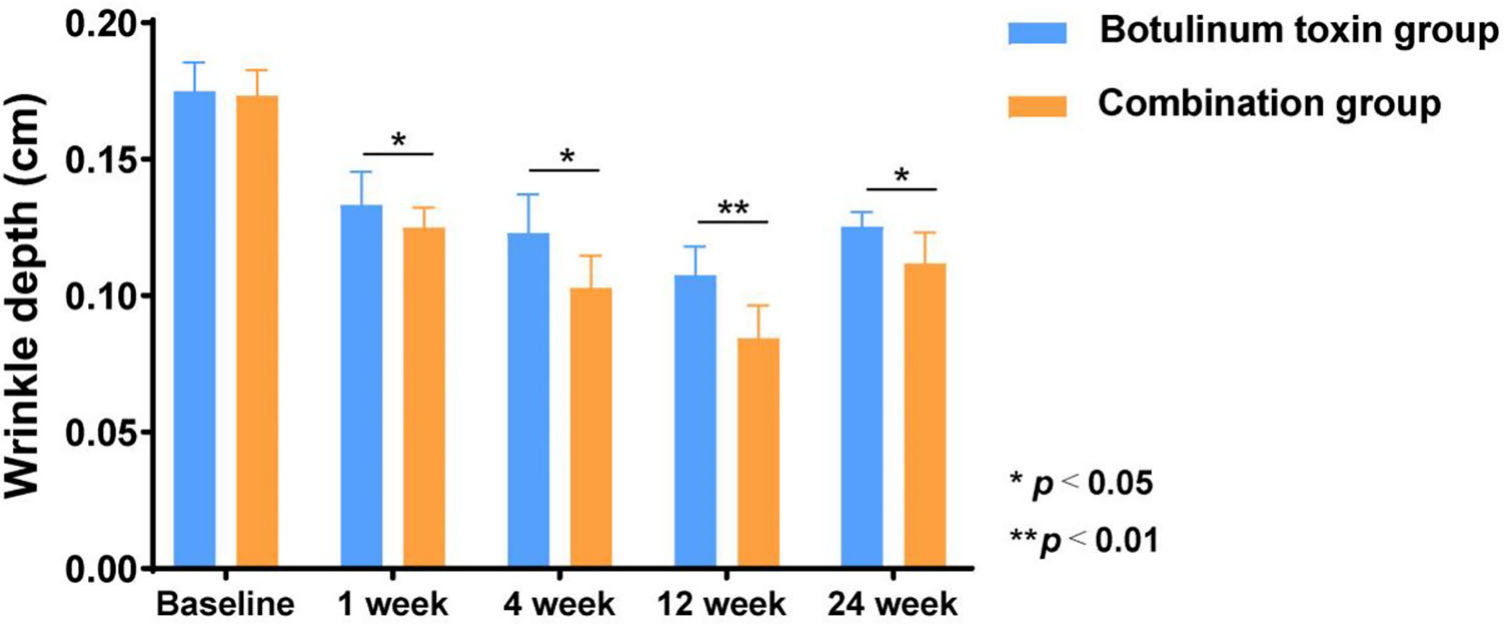
Comparison of wrinkle depth before and after injection

Histograms illustrating changes in wrinkle length and depth revealed that improvements in the observation group were more pronounced than those in the control group. Additionally, the therapeutic effect in the observation group was more stable and lasted longer. At the 6-month follow-up, both groups experienced some deepening and lengthening of wrinkles; however, the change in depth was less pronounced than the change in length (Figs. 6 and 7).

Laboratory results did not reveal any specific abnormalities, and no significant adverse reactions were reported throughout the clinical process.

## Discussion

Among various treatment options available for improving periorbital wrinkles [1], injection therapy has emerged as the preferred approach. Different injection schemes offer distinct advantages, depending on their onset time, mechanism of action, and duration of effect. These therapies can be combined based on the patient’s specific condition to maximize therapeutic outcomes [2, 3].

BTX-A has demonstrated significant efficacy in facial rejuvenation and the treatment of periorbital wrinkles, particularly dynamic wrinkles such as glabellar lines and crow’s feet. However, its effectiveness is limited for gravitation wrinkles, particularly moderate-to-severe static wrinkles [7]. The primary mechanism of BTX-A involves inhibiting acetylcholine release from the presynaptic membrane, thereby relaxing overly contracted muscles to reduce wrinkles and achieve lifting effects. Treatment of dynamic wrinkles with BTX-A typically shows the desired effect within 3–7 days of injection and lasts approximately 3 months. However, increasing the injection dosage in individuals with moderate-to-severe wrinkles often leads to side effects such as stiff facial expressions, excessively raised eyebrow tips, brow ptosis, palpebral edema, difficulty opening eyes, or even blepharoptosis. In recent years, Microbotox has gained popularity for addressing these issues because it helps minimize the side effects, reduce dynamic wrinkles, and achieve a natural facial appearance without stiffness. While this approach improves clinical satisfaction rates for individuals with mild wrinkles, its effectiveness for moderate-to-severe wrinkles remains limited.

The skin contains a high concentration of collagen protein, primarily distributed in the dermis, where it constitutes approximately 75–85% of the total content [8, 9]. Dermal atrophy occurs as collagen fibers within the dermis undergo fragmentation, and the ability to synthesize extracellular matrix components is impaired due to a reduction in fibroblast numbers and the morphological collapse of these cells. This process leads to the formation of wrinkles and depressions. Additionally, the production of extracellular matrix components, such as collagen, hyaluronic acid, and elastin, decreases, whereas the expression of matrix metalloproteinases increases, accelerating the degradation of the extracellular matrix [10] and contributing to the development of static wrinkles. Wrinkles and depressions exhibit significant differences in shape, depth, and length, and such variations can occur even on the same individual’s face. Due to these differences, no single injection material can address all types of skin defects. This limitation highlights why botulinum toxin alone is often insufficient for treating moderate-to-severe static wrinkles effectively in clinical practice. To address this challenge, Zhu [11] evaluated the combined use of BTX-A and hyaluronic acid for reducing facial wrinkles and enhancing skin rejuvenation. Similarly, Kenneth [12] assessed the effects of combining botulinum toxin with hyaluronic acid for rejuvenating specific regions, including the periorbital, temporal, glabellar, and crow’s feet areas. These studies have reported that the combined use of botulinum toxin and hyaluronic acid provides more significant and longer-lasting postoperative effects than using either method alone.

Hyaluronic acid has become the most widely used dermal and subcutaneous filler in clinical practice due to its excellent contour-modifying effects and the availability of a specific resolvase, hyaluronidase. As a biopolymer, collagen is the primary component of animal connective tissue and is the most abundant and widely distributed functional protein in mammals, accounting for 25–30% of the total protein content and exceeding 80% in certain organisms. Compared with hyaluronic acid products, collagen offers distinct advantages for treating periorbital fine lines, which are reflected in several key aspects. First, the collagen injection yields amino acids as metabolic products, which serve as raw materials for collagen synthesis, thereby promoting collagen regeneration and synthesis at the injection site. By contrast, hyaluronic acid yields carbon dioxide and water as metabolites, and collagen production is stimulated locally following its injection. Second, collagen’s dehydration and cohesion properties after injection confer coagulative characteristics, preventing facial overfilled syndrome (FOS), a condition caused by fillers such as hyaluronic acid absorbing water. This results in a more natural appearance. Third, collagen provides precise injection advantages for treating fine lines in delicate areas, such as the periorbital and perioral regions. These advantages have contributed to collagen’s popularity in injection treatments, particularly in China and Japan. Compared with other fillers, collagen products offer the unique benefit of customizable dosage forms, allowing treatments to be tailored based on wrinkle depth. The outcomes appear natural, recovery times are short, and patients experience minimal pain or discomfort at the injection site. Additionally, due to collagen’s coagulability, there is minimal bruising when injected into the superficial to middle layers of the dermis.

In 1981, the FDA approved “Zyderm” as the first injectable cosmetic material, marking a milestone in esthetic medicine. In China, the first medical collagen filling agent, FILLDERM, was approved for sale in 2012 [13]. In this study, we utilized a non-cross-linked, highly purified, specific pathogen-free bovine dermal type I and III atelocollagen infused with lidocaine as the preferred dermal filler. This collagen filling agent is widely used by clinicians in China due to its low immunogenicity, excellent biocompatibility for cell anchoring support, robust mechanical properties from its intact triple-helix structure, and degradability. Its safety and effectiveness have been thoroughly verified through experimental studies. Upon injection, collagen directly supplements type I and III collagen at the treatment site, improving skin texture, reducing static wrinkles, enhancing skin elasticity, improving skin laxity, and stimulating new collagen synthesis, as well as tissue repair and remodeling [14]. In clinical practice, for individuals with moderate-to-severe wrinkles, a modest filling of static wrinkles is performed initially, followed by botulinum toxin injections to induce muscle relaxation for dynamic wrinkles. This combined approach provides immediate effectiveness and prolongs the treatment's effects. The certain limitations of this study is there was a small sample size, this is single-center designed study, and the generalization of the results of the study needs to be considered.

## Conclusion

This clinical trial demonstrated that the observation group, injected with BTX-A along with collagen fillers, exhibited significantly better outcomes and greater satisfaction, which confirms the effectiveness of this treatment. Collagen not only serves as a filling agent but also provides tensile signals to fibroblasts by aiding in the reconstruction of the collagen fiber network. This process stimulates fibroblast activity, promoting collagen regeneration and supporting skin nutritional renewal [15, 16]. Furthermore, the ivory structure of collagen enhances the skin tone and produces noticeable effects through both physical and chemical mechanisms [17]. Beyond wrinkle reduction, this study also emphasized the impact of collagen on skin texture. The results revealed that, one month after treatment, the observation group showed significantly greater improvements in skin elasticity and moisture levels than the control group.

In conclusion, the combination of BTX-A injections and non-cross-linked bovine collagen fillers is an effective approach for treating moderate-to-severe periorbital wrinkles. This method offers minimal trauma, rapid recovery, superior therapeutic outcomes, and higher patient satisfaction.
